# Using Fractionation and Diffusion Ordered Spectroscopy to Study Lignin Molecular Weight.

**DOI:** 10.1002/open.201900129

**Published:** 2019-04-25

**Authors:** James R. D. Montgomery, Priory Bazley, Tomas Lebl, Nicholas J. Westwood

**Affiliations:** ^1^ School of Chemistry and Biomedical Sciences Research Complex University of St Andrews and EaStCHEM St Andrews, Fife KY16 9ST UK

**Keywords:** DOSY NMR, biomass, lignin, molecular weight determination, fractionation

## Abstract

Recent reports demonstrate that applications of the biopolymer lignin can be helped by the use of a fraction of the lignin which has an optimal molecular weight range. Unfortunately, the current methods used to determine lignin's molecular weight are inconsistent or not widely accessible. Here, an approach that relies on 2D DOSY NMR analysis is described that provides a measure of lignin's molecular weight. Consistent results were obtained using this well‐established NMR technique across a range of lignins.

## Graphical abstract

**Evening the playing field**: Using DOSY NMR and the Mark Houwink Sakurada relationship to streamline access to reproducible lignin molecular weight estimations.

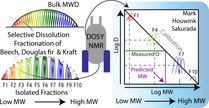

## 1 Introduction

**Figure 1:**
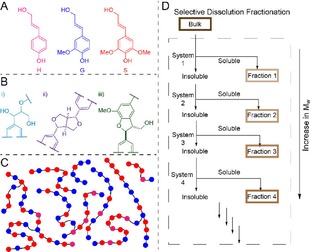
**A** The monolignol phenyl propanoid units that make up lignin: *p*‐coumaryl (H), coniferyl (G) and sinapyl (S) alcohols; **B** the three most abundant linkages present in lignins: i) β‐O‐4, ii) β‐β and iii) β‐5; **C** Representation of a mixture of lignin chains of different lengths and compositions of aromatic units from the monolignols (colour‐coded as in **A**); **D** Schematic representation of a selective dissolution solvent fractionation protocol where lignin material is sequentially stirred in different solvent systems to isolate fractions of different solubility and molecular weights.

Lignin is an abundant biopolymer that has an important role to play in the future of bio‐refineries.^1^,^2^ However, the valorization of lignin remains a challenge due to its complex structure.^3^, ^4^, ^5^, ^6^, ^7^, ^8^, ^9^ Its inherent heterogeneity is a result of at least three contributing factors: i) Lignin is made up of variable amounts of 3 possible C9 monomers (H, G, and S, Figure 1A); ii) different types of linkages are formed by coupling of the C9 monomers, the most abundant of which is the β‐O‐4 linkage (Figure 1B); iii) Lignin has considerably higher variation in chain lengths compared to standard polymers leading to a high polydispersity (2.0–9.0 depending on the source and pretreatment method used, Figure 1C).^10^ It is becoming evident that for any lignin application, discrete regions of the bulk molecular weight distribution (MWD) will be most suitable.^11^,^12^ For example, low molecular weight (MW) Kraft lignin fractions were shown to form thermosets with more optimal properties.^13^ This was determined by first fractionating the bulk material taken directly from the industrial process using a selective dissolution protocol (see Figure 1D). Subsequent conversion of the selected fractions into the corresponding thermosets identified the best fraction to use. From a research point of view, fractionation has also proved to be a vital step in assessing lignin's depolymerisation potential across its MWD. For instance, this approach has been elegantly used to demonstrate that the Lig family of enzymes do in fact process longer lignin chains rather than just di‐ or trimeric lignin‐derived units.^14^ Clearly, lignin MW is an important property to monitor as we progress towards its efficient and complete valorization.

There are numerous approaches for measuring lignin's MW, each with their own limitations. One option is to use MALDI‐TOF MS however, it is challenging to both ionize lignin and ensure uniform ionization across its polydisperse MWD.^15^ Recently, a multiangle laser light scattering (MALLS) method was developed that circumvented issues arising from lignin's florescence when more conventional analyses of this type were performed.^16^

The most popular method used to analyse lignin MW is gel permeation chromatography (GPC).^17^ This excellent technique separates lignin as a function of MW by size exclusion chromatography and the resulting elution profile is calibrated using polymeric standards (e. g. polystyrene).^18^ This technique also provides access to all statistical averages of polymer MWDs the most commonly reported of which are the weight average (M_w_) and number average (M_n_) molecular weights. Although there is wide spread use of this technique, it has been shown that repeat analyses performed on different GPC setups are inconsistent, inhibiting fair comparisons of data across different labs.^19^ Moreover, lignin samples for GPC analysis are usually subject to acetylation to enhance the solubility, although this is not always necessary. If acetylation is required then the GPC analysis can become quite time consuming. Therefore, there is arguably a need for additional analytical approaches. Very recently, IR has been used in combination with multivariate analysis to determine MW and linkage abundances in lignins.^20^

We have previously shown that diffusion ordered spectroscopy (DOSY‐NMR) has potential for use in monitoring lignin MW.^21^ This technique initially measures the diffusivity of molecules in solution and this parameter can then be related to the MW of both small molecules^22^,^23^ and polymers including lignin.^15^,^24^, ^25^, ^26^ In our previous report, diffusion coefficients of lignin fractions of variable MW were linearly correlated with MW data generated via GPC using the Mark‐Houwink‐Sakurada (MHS) relationship.^21^ These fractions were generated by a selective dissolution solvent fractionation that used acetylation to enhance the solubility of Kraft lignin in acetone. It is important to note that the DOSY data was generated using a standard NMR probe, making the method accessible to any lab with a modern NMR spectrometer. The data was processed using standard protocols available within the Topspin 3 and Dynamic Centre 2.5 software packages (the processing of polymer DOSY data has been discussed in detail in a recent review).^27^ All post‐processing of diffusion constants was performed using MS Excel. It should be noted that our earlier study only used acetylated lignin or acetylated model polymers. Therefore we decided to explore whether the correlation we had previously observed between the DOSY‐determined diffusivity and the GPC‐determined molecular weight held for native lignins – that is lignins that had not been acetylated prior to fractionation and NMR analysis. If this were the case, then it is possible that the approach we propose could have much wider scope than we had originally imagined.

The linear scaling of lignin diffusion coefficients found in our and more recently, other^28^ reports, led us to consider the use of MHS analysis to establish a DOSY NMR calibration that would allow quick estimates of lignin MW alongside routine NMR analysis (^1^H NMR and ^1^H,^13^C‐HSQC). To build upon our earlier study, 151 lignin fractions, generated in 13 different fractionations of several types of lignins were analysed. MHS analysis revealed a linear correlation with a reasonable r^2^ value (∼0.9). All DOSY measurements were performed using NMR samples of fixed lignin concentration (86 mg mL^−1^) and temperature 295 K. Several types of lignins were then used to test the concept and showed good agreement between the DOSY derived MW and that measured directly using GPC.

## 2 Results

The study was carried out using three diverse types of lignin (Table S1). An industrial Kraft lignin (ISK, Figure S1), was fractionated twice. Until very recently, it has been difficult to achieve this type of fractionation for unmodified Kraft lignin using volatile organic solvents only.^29^, ^30^, ^31^ In addition, two dioxasolv lignins, one from the softwood, Douglas fir (DFSD) and one from the hardwood, beech (BHD), were prepared using a previously reported method^32^,^33^ (Figures S2 and S3). Several different batches of BHD were prepared using different pretreatment scales. One of the BHD batches was also fractionated and analysed repeatedly to assess reproducibility. The lignins used in this study vary not only in the type of wood and pretreatment used but also in their M_w_ and M_n_ molecular weights, polydispersity, and linkage content (Table S1).

### 2.1 Solvent Optimisation

In order to generate fractions of lignin of variable MW, a solvent fractionation was used to divide the bulk material into narrower MW bands. Whilst our previous acetylation followed by selective dissolution approach enabled the use of volatile organic solvents only, new challenges arose in the further processing of these modified lignins.^34^ Consequently, we decided to focus on the fractionation of unmodified lignins. Recent publications in this area include ultrafiltration,^35^, ^36^, ^37^ selective dissolution^30^,^31^,^38^ and selective precipitation protocols.^29^,^39^,^40^ Of note was the “fast fractionation” reported by Ragauskas,^40^ as it optimized a solvent/co‐solvent system to maximize the amount of soluble kraft lignin using Hilderbrand solubility parameters (for details see Supplementary Information Sections 3 and 4). From this, an experimental solvent optimization was conducted where lignin was stirred in different acetone/methanol (AM) ratios. Yields of the AM soluble material were compared for each type of lignin and for each ratio, giving an optimum of 3 : 2 AM (Tables S2‐S4 and Figure S4). Given the reported protocol followed the selective precipitation approach, we then compared this to a selective dissolution protocol using the same AM solvent system. In brief, whilst the selective precipitation protocol (P2) required less time to complete, the bulk material was more effectively fractionated using the selective dissolution protocol (P1) as this generated fractions of lower and more consistent polydispersity (for more details see Supplementary Information Section 4).

### 2.2 Fractionation and Analysis

**Figure 2:**
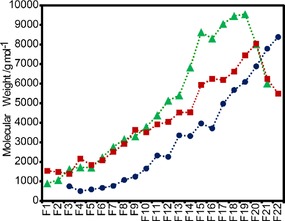
GPC‐determined weight average molecular weights (M_w_) of fractions from fractionations DFL‐1 (Green), BWL‐1 (red) and ISK‐1 (blue).

Inspired by our previous studies,^21^ diethyl ether was used as the anti‐solvent and all three types of lignin were then fractionated using a protocol that started with 100 % diethyl ether and increased by inclusion of 5 % increments of the 3 : 2 acetone‐methanol (AM_3,2_) solution (Figure 1D and Table S6). In all cases, 22 fractions were obtained (fraction 22 refers to the insoluble residual lignin, Figure S6). As expected, yield profiles across the fractions showed considerable differences between lignins (Figure S6 and Tables S6–S8) but the percentage of the starting bulk lignin found in AM_3,2_ soluble fractions 1–21 was high in most cases (Tables S7–S9). In order to collate more independent data points for subsequent studies, DFSD and BHD lignin samples were also fractionated in an analogous way using 9 : 1 and 4 : 1 AM solutions, respectively (Tables S6 and S7). The success of the fractionation protocol was confirmed by GPC analysis showing, with only a few exceptions, that M_w_ values increased gradually with fraction number (Figure 2 and Tables S10–S12).

#### 2.2.1 DOSY NMR Analysis of Lignin Fractions:

DOSY analysis was carried out on selected fractions from 10 different fractionations (Douglas Fir, Beech and Kraft lignins, Tables S13–S15). Importantly, a standard protocol was used for each analysis (see Supplementary Information). The resulting diffusivity values were used in conjunction with the GPC‐determined M_w_ values in a Mark‐Houwink‐Sakurada (MHS)^15^,^21^,^41^ analysis to generate scaling parameters α and log K (Table S16). All MHS analyses resulted in linear Log(D)/Log(M_w_) correlations with reasonably high r^2^ values for all fractionation experiments (e. g. DFL 0.9374; BWL‐1 0.8846 and ISK‐1 0.9265, Table S16). Surprisingly, there was only very small variations in the calculated values of α and log K as a function of the lignin (Table S16). Of note was the similarity of MHS parameters when comparing fractions from unmodified Douglas Fir Softwood lignin with the fractions after they had been acetylated and reanalysed (Table S16, entries 2 and 3). From this comparison, it seems evident that acetylation has only a marginal effect on the structure adopted by lignin chains in a DMSO solution (i. e. modified vs native lignins).

**Figure 3:**
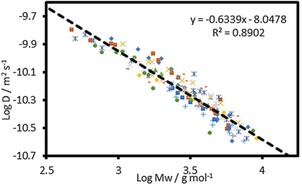
MHS plot made up of 151 combined data points from multiple fractionations of different types of lignins including both unmodified and acetylated fractions. Chart also includes data for G1, G2, KL‐1 and KL‐2 fractionations from our previous report on acetylated model polymer and acetylated Kraft lignin fractionation.^21^ Fractions that generated log D values below −10.6 m^2^ s^−1^ were not included.

The data in Table S16 imply that a generic set of MHS scaling parameters are applicable to a wide range of lignins. Overall MHS analysis based on 151 data points that were obtained in this and previous studies^21^ allows us to propose the generic scaling parameters for lignin of α=−0.63±0.02 and log K=−8.05 m^2^ s^−1^±0.06 when a solution of lignin at 86 mg mL^−1^ in d_6_‐DMSO is analysed at 295 K (Figure 3). These parameters could be used to provide an estimate of M_w_ from the diffusivity of lignins analysed solely by a DOSY‐NMR experiment.

Despite the convincing correlations observed, it was decided to explore in more detail potential limitations. Considering the DOSY NMR component of the analysis, we have previously reported that the heavy fractions, with log D values below −10.6 m^2^ ⋅ s^−1^, exhibited a significantly higher α scaling factor.^21^ This observation was attributed either to the hardware limits of the gradient coil in the standard NMR equipment or to dramatic changes in lignin structure. The latter was further investigated using EPR which showed, in accordance with our hypothesis, that these fractions contained considerably more radicals than the lighter fractions. To minimise systematic errors, all fractions with log D values below the threshold of −10.6 m^2^ s^−1^ were excluded from the correlation in Figure 3 and this approach is not recommended for determining the M_w_ of the heaviest lignin fractions. The standard error of regression (σ) was found to be ±962 g mol^−1^ and the mean absolute percentage error (MAPE) was calculated as 19 % (Table S16, entry 11). The size of error was MW dependent with the greatest uncertainty found towards the heaviest fractions (Figure S7). This could be the result of poor signal to noise in the DOSY experiment or a result of greater degrees of freedom of possible conformations of the larger polymer chains in these fractions. The latter would not be detected by GPC leading to underestimations and possible outliers. Further error analysis was performed to investigate this by removing outliers from the dataset however this did not lead to a significant difference in the α and log K scaling factors, (see Table S16 & Supporting Information Section 10).

Next, potential pitfalls in the GPC determination of the M_w_ data were considered. GPC elution profiles are usually calibrated by well‐defined standards (in our case polystyrene), that are likely structurally different to lignin. For instance, it has been reported that an acetylated hardwood Kraft lignin is more compact than polystyrene in organic solvents (THF and DMSO) and could be approximately 6 times larger than estimated by GPC.^42^ This results from GPC's inability to discriminate between polymers of different shapes/conformations. The plot of measured GPC‐determined M_w_ against M_w_ derived from DOSY for all 151 data points clearly shows that the largest residuals from the identity line occur at high M_w_ (Figure S7). In this region of the M_w_ distribution, there is likely a wider range of possible conformations/shapes of the lignin. Consequently, lignin is more likely to deviate from the structure of polystyrene in THF leading to inaccurate MW measurements. This hypothesis would be supported by the biasing of residuals seen in Figure S8, however, biasing of the MHS scaling factors by branching of larger lignin polymers cannot be excluded as well. We believe this further demonstrates the limitations of the M_w_ determination of lignin using GPC as suggested in the recent literature.^16^

**Figure 4:**
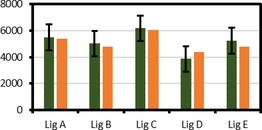
Comparison of molecular weights estimated by DOSY‐NMR (green) and GPC (orange). Error bars indicate the standard error of regression from our generic MHS calibration curve with outliers. For the lignins shown, the GPC derived molecular weight sits significantly within the standard deviation of regression. Lig A and Lig B correspond to separate Douglas fir dioxasolv lignins; Lig C corresponds to a Kraft lignin. Lig D corresponds to a beech butanosolv lignin; Lig E corresponds to formaldehyde stabilised lignin isolated as previously reported.^43^

Despite these likely sources of error, it was decided to test the calibration curve, and hence the overall utility of the proposed DOSY approach, on additional bulk samples of lignins (Figure 4). Therefore, 4 different lignins were prepared and subjected to DOSY NMR analysis. Values for M_w_ were then calculated using the observed diffusivities and our generic MHS scaling parameters. These M_w_ values were then compared with the M_w_ values measured by GPC. Bulk samples of Lig A, Lig B, and Lig C are lignins that are represented in the calibration chart (Figure 3) and showed good agreement with the DOSY derived M_w_ being well within the error of the calibration curve (Figure 4). Bulk samples of Lig D and Lig E (lignins that are not represented in Figure 3) also gave DOSY‐derived M_w_ values within the error of correlation. This illustrates the ability of this method to obtain reliable M_w_ estimations for a wide range of lignins. Therefore, a remarkable match between these two values has been obtained despite the potentially large error associated with the conversion of diffusivity into molecular weight (Figure 4).

In summary, a wide range of lignins were fractionated using a selective dissolution solvent fractionation protocol. A MHS plot containing 151 data points from 13 separate fractionations showed a general trend in lignin diffusivity with increasing MW with an r^2^ value of 0.89. This observation opened the possibility of defining a calibration curve that can be used to deliver a measurement of the molecular weight (M_w_) of lignin samples from diffusivity values measured by DOSY NMR. This approach is potentially a widely accessible method to compare the M_w_ of lignins without GPC analysis that could be utilized by the community of synthetic chemists in addition to the qualitative monitoring of the polymer through processing strategies, in this case fractionation. A potential user only needs to do two things: (i) analyse their lignin sample by DOSY NMR under the conditions we describe and (ii) use our equation to convert the DOSY‐determined diffusivity into an estimate of the molecular weight. Although the proposed method streamlines access to M_w_ of lignin samples, the accuracy of these estimates is likely limited by the reliance on the use of the GPC during the calibration phase and we cannot exclude that there might be occasions when the proposed approach cannot be used. In the future, this may be improved by using more accurate methods to determine the lignin M_w_ for MHS calibration, providing access for the wider community.

## Experimental Section

Full Experimental details of this study can be found in the Supporting Information.

## Conflict of interest

The authors declare no conflict of interest.

## Supporting information

As a service to our authors and readers, this journal provides supporting information supplied by the authors. Such materials are peer reviewed and may be re‐organized for online delivery, but are not copy‐edited or typeset. Technical support issues arising from supporting information (other than missing files) should be addressed to the authors.

Supplementary
