# Supplementary material for: Using Fractionation and Diffusion Ordered Spectroscopy to Study Lignin Molecular Weight
Source: ChemistryOpen. 2019 Apr 25;8(5):601–5. doi: 10.1002/open.201900129 (PMC6511914; doi:10.1002/open.201900129)
Supplement: Supplementary file 1 — Supplementary [file OPEN-8-601-s001.pdf]

Supporting Information

© Copyright Wiley-VCH Verlag GmbH & Co. KGaA, 69451 Weinheim, 2019

Using Fractionation and Diffusion Ordered Spectroscopy to Study Lignin Molecular Weight.

James R. D. Montgomery[+], Priory Bazley[+], Tomas Lebl,* and Nicholas J. Westwood*©2019 The Authors. Published by Wiley-VCH Verlag GmbH & Co. KGaA.
This is an open access article under the terms of the Creative Commons Attribution License, which permits use, distribution and reproduction in any medium, provided the original work is properly cited.

Contents

1. Extracted lignins used in this study 2
2. Exemplar HSQC of lignins used in this study 3
3. Solubility Optimisations for different lignins 5
4. Comparison of Fractionation Procedures using Kraft lignin 9
5. Fractionation of a variety of lignins using Selective dissolution protocol P2 13
6. Gel permeation chromatography data of lignin fractionations 18
7. Diffusion ordered spectroscopy data for lignin fractionations 21
8. Mark-Houwink-Sakurada Scaling parameters of all fractionations 24
9. Comparison of GPC and DOSY derived molecular weights of all 150 data points in the calibration chart 25
10. Statistical analysis of DOSY NMR Calibration 26
11. Experimental 27

References 30

1. Extracted lignins used in this study

Table S1. Characterisation and pretreatments of the bulk lignins used in this study: Indulin Kraft lignin (IKL); large scale Douglas fir dioxasolv lignin (DFD-1_L); small scale (80 g of wood) beech dioxasolv – sample 1 (BHD-1_S), small scale (80 g of wood) beech dioxasolv – sample 2 (BHD-2_S), small scale (80 g of wood) beech dioxasolv – sample 3 (BHD-3_S) and large scale (800 g of wood) beech dioxasolv – sample 1 (BHD-1_L). The weight average (M_w), number average molecular weights (M_n); polydispersity index (PDI); β-O-4, β-β, β-5 and lignin bound Hibbert's ketone (LBHK) content per 100 C_9 units and S:G ratio are given for each lignin. # indicates which fractionation run was used for each lignin. For example, IKL (obtained from Westvaco) was used as received and was fractionated twice (IKL-1 and IKL-2). This material was also used in the solubility studies presented in Tables S2-S4. For details about extraction procedures, see experimental section below.

Extraction	***ISK***	***DFSD(1_L)***	***BHD(1_S)***	***BHD(2_S)***	***BHD(3_s)***	***BHD(1_L)***
M_w	3462	5198	5453	6951	4166	5793
M_n	763	1167	619	310	646	450
PDI	4.5	4.5	8.8	22.4	6.5	12.9
β-O-4	8	13	42	49	38	28
β- β	3	6	17	16	12	14
β-5	2	9	7	6	4	4
LBHK	-	10	4	9	4	4
S:G	-	-	3.5:1	4.2:1	3.7:1	3.6:1
#	ISK-1<br>ISK-2<br>Solubility tests	DFL-1<br>DFL-2<br>DFL-3<br>Solubility tests	BWL-1<br>Solubility tests	Solubility tests	BWL-2	BWL-3<br>BWL-4

2. Exemplar HSQC of lignins used in this study

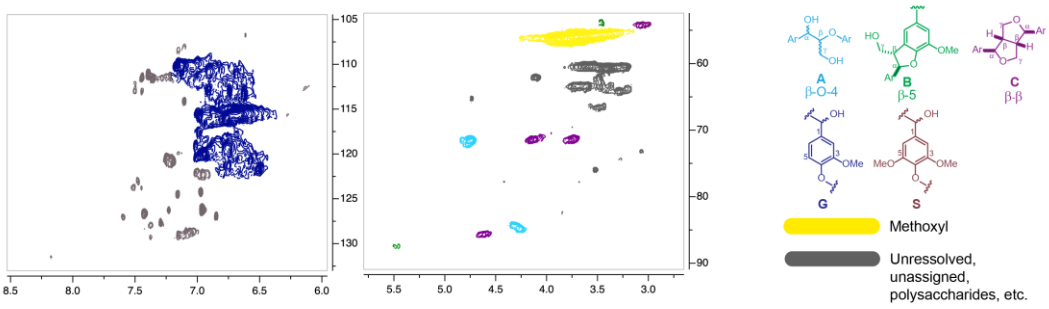

Figure S1: 2D HSQC NMR spectrum of the bulk Indulin Kraft (ISK) lignin used in this study. The spectrum is displayed as the aromatic and linkage regions with relevant cross-peaks coloured according to the key provided.

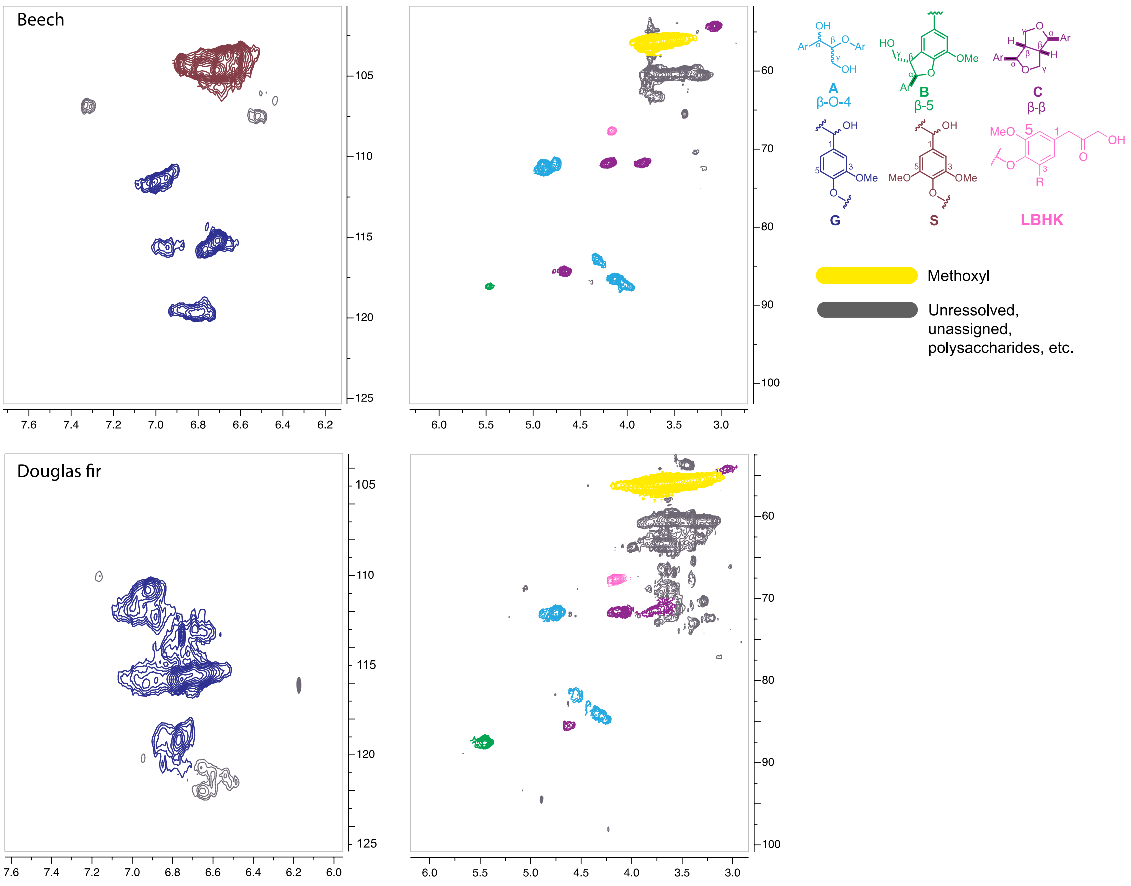

Figure S2: 2D HSQC NMR spectra of the dioxasolv beech (hardwood) lignin and Douglas fir (softwood) lignin used in this study. Both samples were prepared on a small scale (80 g of wood). The spectra are displayed as the aromatic and linkage regions with relevant cross-peaks coloured according to the key provided.

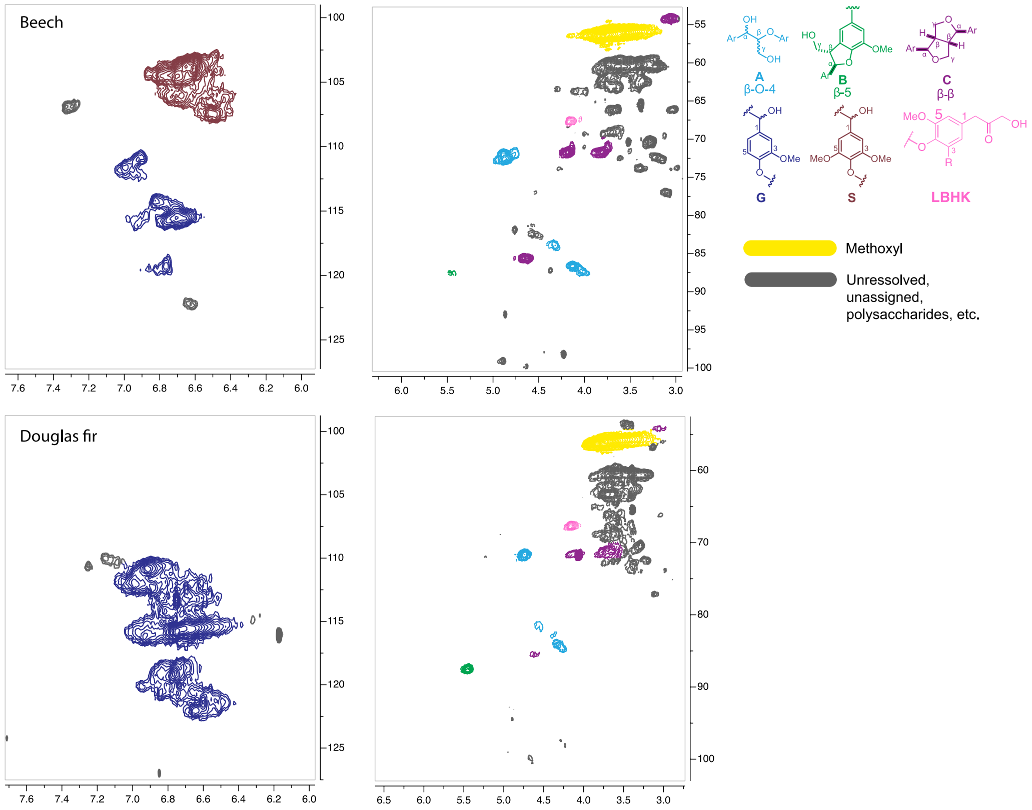

Figure S3: 2D HSQC NMR spectra of the dioxasolv beech (hardwood) lignin and Douglas fir (softwood) lignin used in this study. Both samples were prepared on a large (800 g of wood) scale. The spectra are displayed as the aromatic and linkage regions with relevant cross-peaks coloured according to the key provided.

3. Solubility Optimisations for different lignins

Initially, the solubility of the bulk lignins was explored in a range of 2-component solvent/co-solvent mixtures (Figure S4 & Tables S2-S4). Both dioxasolv lignins showed good solubility (93 – 99%) in a wide range of solvents with different acetone:methanol ratios (Figure S4, 9:1 – 3:2). On the other hand, the solubility of ISK peaked at a 3:2 solvent:co-solvent ratio with a maximum recovered yield of 75% (Figure S4 & Table S4). Since the 3:2 acetone:methanol solvent system seemed to provide optimal solubility for both Kraft and organosolv lignins (Figure S4) it was used in all subsequent fractionations.

Table S2: Solubility optimisation of Douglas Fir softwood dioxasolv lignin - DFSD(1_L). Material was lost during filtration on equipment leading to less than 100% total recovered yield. Only yields of Acetone:Methanol soluble fractions were taken into consideration when comparing solvent ratios.

	**Acetone: MeOH (Solvent/Co-solvent) ratio**														
	**1:0**			**9:1**			**4:1**			**7:3**			**3:2**		
**Initial (g)**	0.3031	0.3069	0.2786	0.3095	0.2987	0.2945	0.2944	0.2960	0.3066	0.2794	0.3052	0.3013	0.3030	0.3067	0.2870
**Soluble (g)**	0.1664	0.1434	0.1136	0.2397	0.2517	0.2583	0.2661	0.2397	0.2756	0.2542	0.2940	0.2836	0.2756	0.2935	0.2736
**Yield (wt%)**	57	48	41	93	97	88	97	91	92	99	98	96	98	98	96
**Average**	49			93			93			98			97		
**Error**	3.8			2.1			1.5			0.7			0.5		
**Total recovered**	0.2927	0.2964	0.2703	0.2581	0.2604	0.2933	0.2734	0.2624	0.2988	0.2566	0.3002	0.2951	0.2807	0.3009	0.2854
**Overall yield (wt%)**	96	97	97	83	87	>99	93	89	97	92	98	98	93	98	99

Table S3: Solubility optimisation of Beech hardwood dioxasolv lignin. Lignin from batches BHD(1_S) and BHD(2_S) was used for each Acetone:Methanol (AM) ratio in this solubility study. Material was lost during filtration on equipment leading to less than 100% total recovered yield. Only yields of AM soluble fractions were taken into consideration when comparing solvent ratios.

	**Acetone: MeOH (Solvent/Co-solvent) ratio**														
	**1:0**			**9:1**			**4:1**			**7:3**			**3:2**		
Initial (g)	0.2518	0.2089	0.2823	0.2264	0.2206	0.3692	0.2336	0.2086	0.3566	0.2696	0.2332	0.3136	0.2409	02056	0.3121
Soluble (g)	0.0257	0.0098	0.0440	0.2204	0.1975	0.3609	0.2334	0.1974	0.3245	0.2666	0.2226	0.2993	0.2282	0.1974	0.3023
Yield (wt%)	11	5	16	97	93	98	>99	96	99	>99	98	>99	>99	97	99
Average	11			96			98			99			98		
Error	2.6			1.2			0.8			0.3			0.5		
Total recovered	0.2269	0.2040	0.2726	0.2261	0.2118	0.3692	0.2335	0.2060	0.3277	0.2667	0.2279	0.2997	0.2283	0.2038	0.3025
Overall yield (wt%)	90	98	97	>99	96	100	>99	99	92	99	98	96	95	99	97

Table S4. Solubility optimisation using Indulin Kraft lignin. Material was lost during filtration on equipment leading to less than 100% total recovered yield. Only yield of Acetone:Methanol soluble fractions were taken into consideration when comparing solvent ratios.

	Acetone: MeOH (Solvent/Co-solvent) ratio																				
	1:0			9:1			4:1			7:3			3:2			1:1			1:4		
Initial (g)	0.3079	0.3333	0.2923	0.3244	0.2730	0.3254	0.3220	0.2744	0.3042	0.3145	0.3420	0.3043	0.3132	0.3458	0.3013	0.3005	0.2354	0.2722	0.3515	0.3191	0.2857
Soluble (g)	0.0752	0.0650	0.0635	0.1091	0.1060	0.1477	0.1799	0.1128	0.1568	0.2255	0.2172	0.1770	0.2401	0.2481	0.2013	0.2361	0.1556	0.1845	0.2388	0.1822	0.1566
Yield (wt%)	25	21	22	43	40	46	62	42	52	76	66	64	82	73	68	81	66	70	69	58	58
Average	23			43			52			69			75			72			62		
Error	1.0			1.4			4.7			3.0			3.3			3.7			3.0		
Total recovered	0.3008	0.3168	0.2854	0.2540	0.2668	0.3199	0.2913	0.2681	0.3007	0.2963	0.3289	0.2770	0.2919	0.3376	0.2974	0.2912	0.2024	0.2641	0.3458	0.3163	0.2697
Overall yield (wt%)	98	95	98	78	98	98	91	98	99	94	96	91	93	98	99	97	86	97	98	99	94

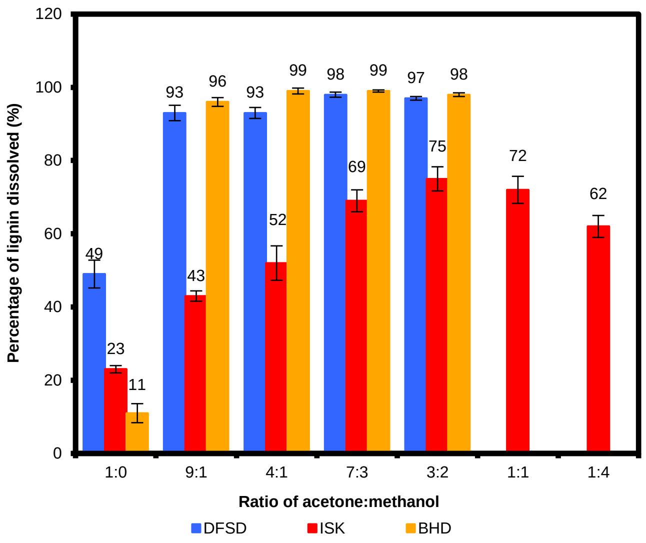

Figure S4. Solubility tests performed for all three sources of lignin, DFSD, ISK and BHD lignins using different ratios of acetone:methanol. Bar labels correspond to average percentage yield of soluble material for each solvent system. Error bars correspond to the standard deviation of the percentage yield of soluble fractions for each type of lignin.

4. Comparison of Fractionation Procedures using Kraft lignin

In a very recent report,[S1] a "fast fractionation" protocol has been reported that uses a 7:3 acetone:methanol ($AM_{7,3}$) solvent:co-solvent system. This ratio was reached by initially calculating Hilderbrand solubility parameters (δ-values) for single solvents and mixtures of solvents. The 7:3 acetone/methanol system was selected because it had a similar δ value to lignin.[S1,S2] In this previous protocol, a sample of Kraft lignin was dissolved in the $AM_{7:3}$ solution (leaving some insoluble material behind). Adding hexane sequentially to the filtered $AM_{7:3}$ solution led to the precipitation of lignin fractions with decreasing molecular weight. Considering the complementary nature of this protocol and our protocol, it was decided to compare both approaches. For each protocol, 8 lignin fractions were prepared from the same batch of Kraft lignin starting material (using an initial mass of 20 g of lignin, Table S4 and experimental). The results were then compared.

Table S4 Recovered yields from fractionation protocol comparison. P1 corresponds to the protocol reported by us in this paper. P2 corresponds to fast fractionation protocol previously reported in the literature.[S1] Both protocols used an initial mass of Kraft lignin of 20 g. Numbers in parentheses indicates order of isolation of each fraction for the respective protocol. *Order of fractions from selective precipitation protocol (P2) reversed to enable easy comparison with fractions from selective dissolution protocol (P1).

Row	Reported Fraction Numbers	P1 (g)	P2* (g)
**1**	**F1**	1.224 (1st)	3.3664 (8th)
**2**	**F2**	2.3395 (2nd)	0.7072 (7th)
**3**	**F3**	2.749 (3rd)	1.4099 (6th)
**4**	**F4**	3.0971 (4th)	1.9431 (5th)
**5**	**F5**	1.5011 (5th)	1.3344 (4th)
**6**	**F6**	2.1324 (6th)	2.7362 (3rd)
**7**	**F7**	1.6314 (7th)	2.8326 (2nd)
**8**	**F8**	3.6941 (8th)	4.6387 (1st)

The fraction yield profile from our selective dissolution protocol (now referred to as P1) showed a nearly Gaussian distribution of masses for fractions F1-F7 with the exception of F5 (Figure S5A). However, the largest proportion of material was in the insoluble fraction F8 (18 wt%, Figure S5A). The yield profile of the previously reported selective precipitation protocol (now referred to as P2) showed a different trend. Whilst the insoluble fraction F8 was again the largest by recovered mass (23 wt%), the mass of material steadily increased between F2 (4 wt%) to F7 (14 wt%, except for F5). Furthermore, F1 gave the second highest yield for the P2 fractionation at 17 wt%. This F1 fraction corresponds to the material that remains soluble in an $AM_{3,2}$ /hexane (1:1) solution at the end of P2. The large amount of material present in F1 demonstrates that it is difficult to achieve the precipitation of low molecular weight material from solution using P2. In contrast in P1, it is relatively easy to dissolve the low molecular weight material selectively from the bulk solid phase. This inability to isolate the low molecular weight lignin chains could potentially prove a limitation of protocol P2.

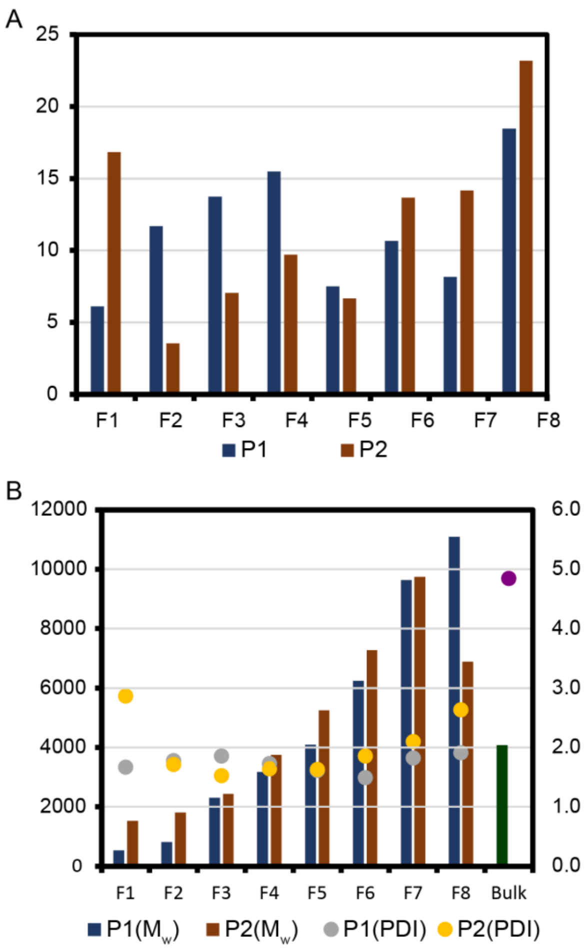

Figure S5. A: Fraction yield profiles from selective dissolution fractionation protocol (P1 - blue bars) and selective precipitation fast fractionation protocol (P2 - brown bars). Yields are given as wt% of the initial mass of Indulin Kraft lignin (20 g). Fractions have been ordered from lowest molecular weight to highest molecular weight for both fractionations (F1-F8) to enable easy comparison. For P1, fractions are isolated in the order of lowest to highest. For P2, fractions are collected highest MW to lowest MW (see experimental). **B:** GPC data (M_w and PDI) of the fractions obtained by using selective dissolution protocol (P1 - blue bars and grey circles, respectively) and the previously reported selective precipitation fast fractionation protocol[1] (P2 – brown bars and yellow circles, respectively). Bulk values of M_w (green bar) and PDI (purple circle) have been included for comparison. NB: Fractions from P2 have been reordered.

The GPC-determined weight average molecular weight (M_w) values for F1-F7 resulting from our fractionation protocol (P1) were consistently lower than the corresponding values obtained for the fast fractionation protocol P2 (Figure S5B and Table S5). However, the same upward trend in M_w was shown for both protocols (Figure S5B, blue and brown bars). A divergence in M_w values was found for the insoluble fractions (F8). P1 yielded an insoluble fraction (F8) that followed the upward trend in M_w observed for F1-F7. On the other hand, F8 from P2 showed a considerable decrease in M_w relative to F7 (Table S5). This discrepancy can be rationalised if both polydispersity index (PDI) and yields of the insoluble fractions are taken into account. P1-F8 has a lower yield (Figure S6) and lower PDI (18 wt% and 1.9 respectively) compared to P2-F8 (23 wt% and 2.6 respectively). Therefore, it seems that the main difference between the P1 and P2 fractionation protocols is the presence of low molecular weight species in the insoluble fraction. F8 is isolated either at the end or the beginning of the fractionation protocol P1 and P2, respectively. For P2, the presence of lower molecular weight chains may inhibit the dissolution of the higher molecular weight chains in the bulk that can result in a higher yield of a more polydisperse insoluble fraction F8. In addition, the PDI's of fractions from P1 are more consistent with an average of 1.7 ± 0.1, compared to 2.0 ±0.5 for P2, suggesting a more uniform separation of material across 8 fractions and a more effective fractionation.

Table S5. GPC data from fractionations using selective dissolution fractionation protocol P1 and selective precipitation fast fractionation protocol P2. Weight average (M_w), number average (M_n) and polydipersity (PDI) is given for each fraction.

	Frac. No.	P1 M_w (g mol^{-1})	P1 M_n (g mol^{-1})	P1 PDI	P2 M_w (g mol^{-1})	P2M_n (g mol^{-1})	P2 PDI
**1**	**F1**	533	319	1.7	1525	532	2.9
**2**	**F2**	810	455	1.8	1804	1052	1.7
**3**	**F3**	2304	1242	1.9	2430	1596	1.5
**4**	**F4**	3179	1840	1.7	3742	2282	1.6
**5**	**F5**	4085	2533	1.6	5247	3223	1.6
**6**	**F6**	6243	4183	1.5	7281	3921	1.9
**7**	**F7**	9634	5281	1.8	9744	4638	2.1
**8**	**F8**	11098	5801	1.9	6892	2618	2.6

Nevertheless, protocol P2 did show some benefits over P1. The total time to complete the process was shorter (3 hours compared to 15 hours) and P2 used a smaller total volume of organic solvents (400 mL compared to 1600 mL). Both protocols used volatile flammable solvents (Et_2O in P1 and hexanes in P2) which would raise issues on upscaling such a process to industrial levels. CPME is seen as a greener solvent however, would not necessarily be a suitable anti-solvent, particularly when trying to precipitate polymers from a solution. Overall, we believe both fractionations produced comparable results as both generated fractions with PDI's considerably smaller than the bulk material (PDI of 4.8).

5. Fractionation of a variety of lignins using Selective dissolution protocol P2

Table S6 $AM_{3:2}$: diethyl ether solvent ratios (v/v) of solvent systems used to isolate each fraction in the new selective dissolution protocol of unprotected lignins. Ten volumes (v/w) of solvent based on the initial mass of lignin was used at each step of the fractionation process.

Fraction	$AM_{3,2}$: Diethyl Ether Ratio
F1	0:100
F2	5:95
F3	10:90
F4	15:85
F5	20:80
F6	25:75
F7	30:70
F8	35:65
F9	40:60
F10	45:55
F11	50:50
F12	55:45
F13	60:40
F14	65:35
F15	70:30
F16	75:25
F17	80:20
F18	85:15
F19	90:10
F20	95:5
F21	100:0
F22	Residue

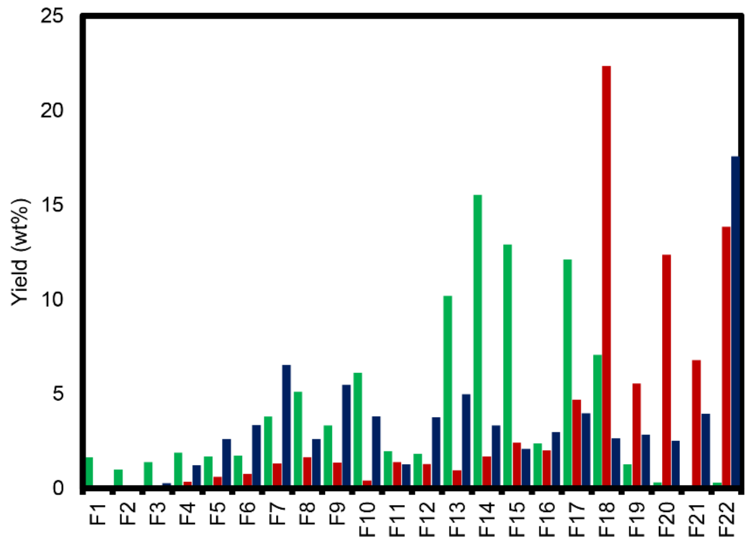

Figure S6. Fraction yield profiles of DFSD(1_L) (fractionation DFL-1 (green)), BHD(1_S) (fractionation - BWL-1 (red)) and ISK (fractionation ISK-1 (blue)). Yields are reported as a wt%. Fractions F1-F21 corresponds to soluble $AM_{3:2}$ fractions. Fraction F22 corresponds to residual lignin at the end of the fractionation process.

Table S7: Recovered yields (g) using DFSD(1_L) (fractionation DFL-1, F1-F22). Amount of acetone:methanol (AM) solutions used to isolate each fraction is given. Subscript numbers in fractionation labels indicate the acetone/methanol (solvent/co-solvent) ratio used in the fractionation. Initial mass of DFSD(1_L) used in each fractionation is given in row 1. Amounts are given as a mass of soluble material obtained from each step of the fractionation (F2-F24). Row 23 indicates the total amount of the AM soluble lignin from the fractionation. Row 24 indicates the amount of AM insoluble material from each fractionation. Data in row 25 indicate the total amount of lignin recovered in the fractionation. Fractionation of DFSD(1_L) using a 9:1 AM solution was carried out in duplicate (fractionations DFL-2 & DFL-3).

	Fraction Number	Amount of AM Solution (%)	DFL-$1_{3,2}$ (g)	DFL-$2_{9,1}$ (g)	DFL-$3_{9,1}$ (g)
1	Bulk	-	10.158	10.235	9.940
2	F1	0	0.166	0.099	0.0902
3	F2	5	0.101	0.090	0.0704
4	F3	10	0.140	0.113	0.0909
5	F4	15	0.192	0.060	0.1163
6	F5	20	0.171	0.059	0.2053
7	F6	25	0.176	0.153	0.1741
8	F7	30	0.385	0.179	0.123
9	F8	35	0.518	0.175	0.1494
10	F9	40	0.338	0.238	0.2412
11	F10	45	0.619	0.277	0.1542
12	F11	50	0.200	0.171	0.2943
13	F12	55	0.186	0.341	0.2879
14	F13	60	1.034	0.204	0.3463
15	F14	65	1.577	0.283	0.1734
16	F15	70	1.310	0.309	0.2705
17	F16	75	0.240	0.349	0.3311
18	F17	80	1.229	0.526	0.571
19	F18	85	0.717	0.386	0.1795
20	F19	90	0.128	0.135	0.1217
21	F20	95	0.032	0.230	0.2336
22	F21	100	0.003	0.193	0.2251
23	Yield F1- F21 (g)		9.460	4.568	4.449
24	F22 (Insoluble Fraction)		0.030	1.803	2.473
25	Yield F1-F22 (g)		9.490	6.461	6.922
26	Total Yield (%)		93	63	70

Table S8. Recovered yields from Beech hardwood lignin (BHD) fractionations (BWL-1 to 4, F1-F22). Amount of acetone:methanol (AM) solutions used to isolate each fraction is given. Subscript numbers in fractionation labels indicate the acetone:methanol (solvent:co-solvent) ratio used in the fractionation. Initial mass of BHD used in each fractionation is given in row 1. Yields are given as a mass of soluble material obtained from each step of the fractionation (F2-F22). Row 23 indicates the total amount of the AM soluble lignin from the fractionation. Row 24 indicates the amount of AM insoluble material in each fractionation. Data in row 25 indicate the total amount of material recovered during the fractionation. Fractionation of BHD using a 3:2 AM solution was carried out in triplicate (BWL-1 using BDH(1_S), BWL-2 using BDH(2_S) & BWL-3 using BDH(1_L)). BWL-4 used lignin from batch BDH(1_L) with the modified AM ratio as indicated.

	Fraction Number	Amount of AM Solution (%)	BWL-$1_{3,2}$ (g)	BWL-$2_{3,2}$ (g)	BWL-$3_{3,2}$ (g)	BWL-$4_{4,1}$ (g)
**1**	**Bulk**	**-**	7.740	9.769	50.144	9.240
**2**	**F1**	**0**	0.007	0.008	0.042	0.014
**3**	**F2**	**5**	0.004	0.011	0.098	0.017
**4**	**F3**	**10**	0.011	0.016	0.206	0.038
**5**	**F4**	**15**	0.027	0.032	0.332	0.062
**6**	**F5**	**20**	0.047	0.068	0.811	0.104
**7**	**F6**	**25**	0.058	0.121	0.505	0.138
**8**	**F7**	**30**	0.102	0.193	0.368	0.103
**9**	**F8**	**35**	0.127	0.238	0.126	0.103
**10**	**F9**	**40**	0.105	0.266	0.595	0.143
**11**	**F10**	**45**	0.031	0.218	1.761	0.181
**12**	**F11**	**50**	0.107	0.193	0.635	0.139
**13**	**F12**	**55**	0.099	0.167	0.794	0.061
**14**	**F13**	**60**	0.074	0.175	1.006	0.073
**15**	**F14**	**65**	0.131	0.188	1.172	0.127
**16**	**F15**	**70**	0.186	0.474	4.321	0.181
**17**	**F16**	**75**	0.155	0.260	4.920	0.151
**18**	**F17**	**80**	0.362	0.433	8.779	0.669
**19**	**F18**	**85**	1.730	0.601	5.512	0.536
**20**	**F19**	**90**	0.428	0.761	2.601	1.480
**21**	**F20**	**95**	0.956	0.473	3.552	1.278
**22**	**F21**	**100**	0.525	0.585	0.735	0.387
**23**	**Yield F1- F21 (g)**		5.271	5.480	38.871	5.985
**24**	**F22 (Insoluble Fraction)**		1.071	2.849	11.196	2.745
**25**	**Yield F1-F22 (g)**		6.342	8.329	50.067	8.730
**26**	**Total Yield (%)**		82	85	>99	94

Table S9. Recovered yields from Indulin Kraft Lignin (ISK) (fractionations ISK-1 and ISK-2 F1-F22). Amount of acetone:methanol (AM) solutions used to isolate each fraction is given. Subscript numbers in fractionation labels indicate the acetone:methanol (solvent:co-solvent) ratio used in the fractionation. Initial mass of ISK used in each fractionation is given in row 1. Yields are given as a mass of soluble material obtained from each step of the fractionation (F2-F24). Row 23 indicates the total amount of the AM soluble lignin from the fractionation. Row 24 indicates the amount of AM insoluble material in each fractionation. Data in row 25 indicate the total amount of material recovered during the fractionation. All fractionations of ISK lignin used a 3:2 AM solution ratio.

	**Fraction Number**	**Amount of AM Solution (%)**	**ISK-1$_{3,2}$ (g)**	**ISK-2$_{3,2}$ (g)**
**1**	**Bulk**		10.740	10.130
**2**	**F1**	**0**	0.010	0.009
**3**	**F2**	**5**	0.014	0.091
**4**	**F3**	**10**	0.029	0.076
**5**	**F4**	**15**	0.132	0.119
**6**	**F5**	**20**	0.279	0.195
**7**	**F6**	**25**	0.359	0.230
**8**	**F7**	**30**	0.700	0.353
**9**	**F8**	**35**	0.280	0.166
**10**	**F9**	**40**	0.588	0.420
**11**	**F10**	**45**	0.409	0.094
**12**	**F11**	**50**	0.136	0.182
**13**	**F12**	**55**	0.402	0.456
**14**	**F13**	**60**	0.534	0.410
**15**	**F14**	**65**	0.357	0.254
**16**	**F15**	**70**	0.224	0.101
**17**	**F16**	**75**	0.320	0.096
**18**	**F17**	**80**	0.425	0.309
**19**	**F18**	**85**	0.284	0.190
**20**	**F19**	**90**	0.304	0.494
**21**	**F20**	**95**	0.269	0.146
**22**	**F21**	**100**	0.423	0.095
**23**	**Yield F1- F21 (g)**		6.477	4.485
**24**	**F22 (Insoluble Fraction)**		1.887	0.447
**25**	**Yield F1-F22 (g)**		8.365	4.932
**26**	**Total Yield (%)**		78	49

6. Gel permeation chromatography data of lignin fractionations

Table S10. GPC analysis of fractions from fractionation of batch DFSD(1_L). For experimental of GPC analysis, see section 10.

	*M_w (g mol^{-1})			M_n(g mol^{-1})		
	**DFL-1 #**	**DFL-2**	**DFL-3**	**DFL-1**	**DFL-2**	**DFL-3**
Bulk	5198	-	-	1167	-	-
F1	896	896	478	425	512	79
F2	1070	1376	643	487	599	80
F3	1629	1917	673	690	773	106
F4	1716	1539	896	757	624	98
F5	1702	1623	1082	606	842	133
F6	2229	1992	920	1029	996	129
F7	2760	2349	1745	1096	1247	808
F8	3163	2612	1907	1463	1311	1009
F9	3301	2907	2371	1286	1573	1282
F10	3786	3387	2629	1607	1889	1277
F11	4378	3687	3212	1431	1295	1709
F12	5133	4139	3079	1780	1917	1237
F13	5402	4881	2950	1633	1960	1411
F14	6831	4649	3775	1755	1688	1636
F15	8631	4866	4464	1573	1701	1934
F16	8305	4850	4757	673	1795	2118
F17	9049	5886	5143	932	1994	1717
F18	9463	5924	5877	918	1106	1809
F19	9555	4368	5711	544	639	1709
F20	8011	6181	6147	974	1087	1819
F21	6010	7998	6392	475	2047	1478

* data plotted in Figure 2

Data plotted in Figure S6

Table S11. GPC analysis of fractions from fractionation of beech hardwood lignin

	*M_w (g mol^{-1})				M_n (g mol^{-1})			
	**BWL-1 #**	**BWL-2**	**BWL-3**	**BWL-4**	**BWL-1**	**BWL-2**	**BWL-3**	**BWL-4**
Bulk	6951	5453	5793	5793	310	619	450	450
F1	1539	2105	902	1204	281	845	330	370
F2	1479	1692	1169	1045	348	614	339	351
F3	1381	1670	1256	1396	562	771	423	505
F4	2159	1540	1634	1396	847	849	718	470
F5	1840	1619	1921	1747	875	977	749	760
F6	2080	1717	1544	1738	981	983	538	856
F7	2512	1853	2163	1842	1284	1223	969	816
F8	2917	2053	2291	2042	1390	1407	876	853
F9	3631	2346	2535	2310	1591	1590	893	1076
F10	3516	2823	2782	2703	839	1664	1118	1131
F11	3918	2962	3230	3040	1592	1883	970	1372
F12	4044	3744	3501	3290	1367	1800	1025	1003
F13	4522	3489	3794	3847	2747	1920	1157	2220
F14	4536	4060	4446	4160	1282	1638	1549	2716
F15	5936	4027	5437	4833	1829	2351	1837	2735
F16	6244	4291	6016	4946	1876	2120	1101	2751
F17	6189	4848	6462	5421	1364	2694	1152	2796
F18	6617	5293	9038	6499	1848	2310	1607	1998
F19	7451	5975	9079	8187	3737	2901	1293	1521
F20	8042	6217	9313	9175	842	1964	934	1402
F21	6247	6742	9054	8499	734	3220	851	887
F22 (Insoluble)	5486	8438	7720	6799	590	1016	276	675

* data plotted in Figure 2

Data plotted in Figure S6

Table S12. GPC analysis of fractions from fractionation of Indulin Kraft lignin

	*M_w (g mol^{-1})		M_n (g mol^{-1})	
	**IKL-1 #**	**IKL-2**	**IKL-1**	**IKL-2**
Bulk	3462	3462	763	763
F1	-	307	-	129
F2	-	596	-	328
F3	746	879	394	388
F4	501	770	302	362
F5	587	534	346	297
F6	670	691	357	365
F7	772	969	429	442
F8	1074	1074	533	644
F9	1237	1298	707	497
F10	1658	1526	965	468
F11	2325	1628	1083	678
F12	2254	1749	1112	675
F13	3349	2204	1517	953
F14	3314	2584	1461	841
F15	3966	3081	1573	1090
F16	3709	4213	1269	2865
F17	4973	4360	1436	324
F18	5675	4046	1570	558
F19	6090	3625	3522	641
F20	6885	8674	1445	2388
F21	7788	9046	2211	1732
F22 (Insoluble)	8381	8840	651	807

* data plotted in Figure 2

Data plotted in Figure S6

7. Diffusion ordered spectroscopy data for lignin fractionations

Table S13. Diffusion coefficients ($m^2\ s^{-1}$) measured by DOSY-NMR of a selection of fractions from the fractionations of Douglas Fir Softwood Dioxasolv (DFSD) lignin obtained using DOSY analysis of a selection of fractions. DFL-2(Ac) corresponds to acetylated lignin fractions from DFL-2 fractionation. Only a few fractions were chosen to compare MHS parameters of acetylated and non-acetylated samples.

	DFL-1	DFL-2	DFL-2(Ac)	DFL-3
F1		-9.86		-9.80
F2	-9.90	-9.93		-9.87
F3	-10.03	-10.03		-9.91
F4	-10.03	-9.99		-9.90
F5	-10.11	-10.00		-9.99
F6	-10.08	-10.00		-9.91
F7	-10.16	-10.08		-9.97
F8	-10.25	-10.19	-10.13	-10.02
F9	-10.17	-10.21	-10.22	-10.11
F10	-10.33	-10.25	-10.26	-10.14
F11	-10.28	-10.32	-10.30	-10.16
F12	-10.32	-10.35		-10.28
F13	-10.42	-10.40		-10.31
F14	-10.46	-10.38	-10.37	-10.34
F15		-10.44	-10.37	-10.37
F16		-10.44		-10.40
F17		-10.51		-10.30
F18		-10.41		-10.48
F19		-10.54		-10.51
F20		-10.60		
F21		-10.50		
F22 (insoluble)				

Table S14. Diffusion coefficients (m^2 s^{-1}) measured by DOSY-NMR of a selection of fractions from the fractionations of Beech Hardwood Dioxasolv (BHD) lignin.

	BWL-1	BWL-2	BWL-3	BWL-4
F1	-	-	-	-
F2	-	-	-10.0768	-
F3	-	-	-	-
F4	-	-	-10.1427	-10.1216
F5	-	-10.1752	-	-
F6	-	-	-	-
F7	-10.2798	-	-10.1713	-10.1533
F8	-10.3468	-10.1805	-	-
F9	-10.3429	-10.0904	-	-
F10	-	-10.1146	-10.2027	-10.224
F11	-10.4023	-10.1649	-	-
F12	-10.3401	-10.2182	-10.3439	-10.2958
F13	-10.4776	-10.3429	-	-
F14	-10.4473	-10.3382	-10.2916	-
F15	-10.5346	-10.2076	-	-
F16	-10.5544	-	-	-
F17	-10.4776	-	-10.5622	-10.3872
F18	-	-	-	-
F19	-10.5918	-10.4283	-	-
F20	-	-	-	-10.5732
F21	-	-10.4157	-	-
F22 (insoluble)	-	-	-	-

Table S15. Diffusion coefficients (m^2 s^{-1}) measured by DOSY-NMR of a selection of fractions from the fractionations of Indulin Kraft (ISK) lignin obtained using DOSY analysis of a selection of fractions. Missing datapoints due to fractions that contained insufficient material to make an NMR sample of the required concentration.

	ISK-1	ISK-2
F1		
F2		
F3		
F4		-9.958607315
F5	-9.8569852	
F6	-9.829738285	-9.910094889
F7	-9.872895202	-9.958607315
F8	-9.928117993	-9.954677021
F9	-9.962573502	-9.995678626
F10	-10.01863449	-10.10734897
F11	-10.09474395	-10.12262865
F12	-10.18111459	-10.21752738
F13	-10.22329882	
F14	-10.11125904	-10.30189945
F15	-10.22988471	
F16	-10.24488773	
F17	-10.23807216	-10.53610701
F18	-10.27818938	
F19	-10.4424928	
F20	-10.39794001	-10.5214335
F21	-10.52432881	
F22 (Insoluble)	-10.57348874	

8. Mark-Houwink-Sakurada Scaling parameters of all fractionations

Table S16. MHS parameters measured from selected fractionation experiments (entries 1-10). MHS parameters measured by combining 150 data points from all fractionation experiments in this study (entry 11). *Entry 12 corresponds to the same data points as in entry 11 minus 12 outliers. All fractions with log D values below -10.6 $m^2\ s^{-1}$ were excluded from both calibration charts in entries 11 and 12. Standard error of regression (σ), mean absolute percentage error (MAPE), average error and average relative error is given for calibration charts in entries 11 and 12.

Entry	Fractionation	α	Log K	R^2	σ $g\ mol^{-1}$	MAPE (%)	Average Error ($g\ mol^{-1}$)	Average Relative error (%)
**1**	DFL-1	--0.67	-7.88	0.9374				
**2**	DFL-2	-0.85	-7.27	0.926				
**3**	DFL-2(Ac)	-0.82	-7.35	0.9449				
**4**	DFL-4	-0.61	-8.12	0.9057				
**5**	BWL-1	-0.65	-8.06	0.8846				
**6**	BWL-2	-0.51	-8.46	0.6426				
**7**	BWL-3	-0.59	-8.23	0.8627				
**8**	BWL-4	-0.55	-8.36	0.976				
**9**	IKL-1	-0.58	-8.18	0.9265				
**10**	IKL-2	-0.66	-8.02	0.9216				
**11**	combined plot	-0.63	-8.05	0.8902	962	19	128	-3
**12**	combined plot*	-0.60	-8.15	0.9019	698	17	-86	-2

9. Comparison of GPC and DOSY derived molecular weights of all 150 data points in the calibration chart

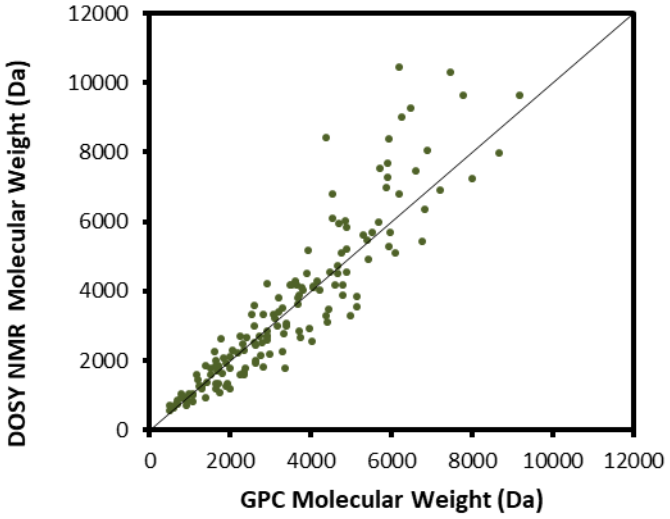

Figure S7. Comparison of molecular weight data measured by GPC and DOSY NMR. Identity line shown to highlight discrepancies between molecular weight measurements. Largest residuals appear in high molecular weight region where lignin chains are likely to adopt more contrasting shapes/conformation when compared to the polystyrene standards used in the GPC analysis.

10. Statistical analysis of DOSY NMR Calibration

Inspection of the residual plot of relative errors showed a largely random distribution with a possible slight bias for a few outliers that result in an underestimation (blue series, Figure S8). This constitutes a possible systematic error. Removing outliers from the data set changed the α and log K scaling factors (to -0.60 & -8.15 g mol^{-1} respectively) and reduced both σ to ± 698 g mol^{-1} and MAPE to 17%. Inspection of the new residual plot of relative errors showed the biasing seen before (blue series, Figure S8) had been reduced (orange series, Figure S8 and Table S16) but was still present. Other sources of error were then considered.

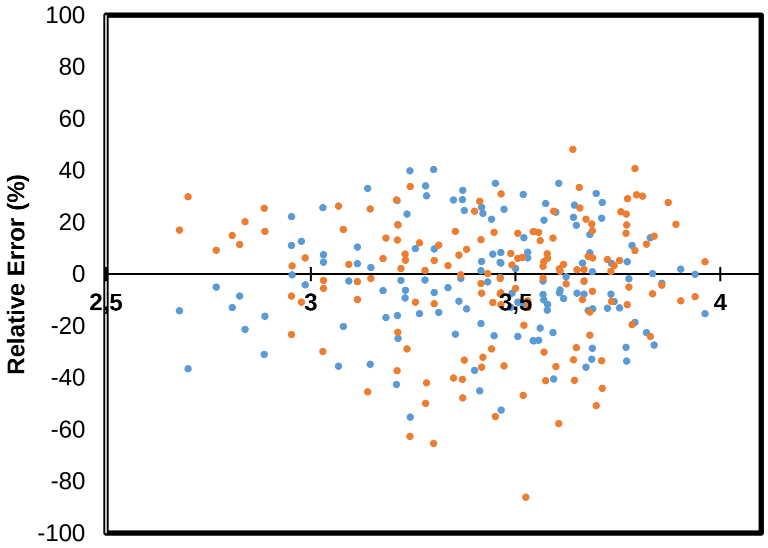

Figure S8. Residual plot of relative errors from the calibration chart in Figure 3 main manuscript (blue series) and the same dataset with outliers removed (orange series), plotted against the M_w (g mol^{-1}), measured directly from the GPC ($M_{w(GPC)}$). Relative errors were calculated using R.E.=((($M_{w(GPC)}$-$M_{w(D)}$)/$M_{w(GPC)}$) x 100). Negative relative errors correspond to when $M_{w(D)}$>$M_{w(GPC)}$.

11. Experimental

General Procedures

Chemical reagents were obtained from Sigma-Aldrich, Fischer Scientific and Acros organics and were used as received unless specified. Indulin Kraft lignin was sourced from Ingevity Corporation. Beech wood shavings and Douglas fir sawdust was purchased from Hot Smoked ltd.

NMR analysis

^{1}H NMR was performed on a Bruker Avance III 700 MHz spectrometer equipped with a nitrogen cooled cryoprobe (Prodigy). The residual solvent peak was used as an internal standard. DOSY experiments were performed using the ledbpgp2s pulse sequence. Gradient amplitude (6.56 G.mm-1) was calibrated using the residual signal of HDO in D2O. The diffusion delay (Δ) and gradient pulse length (δ) were optimized for each sample using the 1D DOSY experiment with the ledbpgp2s1d pulse sequence in order to achieve ca. 5-10% residual signal at 98% gradient strength (compared to 10% gradient strength). Each pseudo-2D experiment consisted of series of 32 spectra acquired with 65536 data points. The gradient pulses were incremented from 10% to 98% with a linear ramp. The temperature was set and maintained at 295 K. Data sets were processed by Fourier transformation in F2, using line broadening of 10 Hz, followed by a baseline correction. The DOSY analysis was then performed in Bruker Dynamics Center 2.3. Manual peak picking was performed for each dataset and peak intensities were used to measure the signal decay. Error estimation of the fit was performed at the 95% confidence level. All samples were prepared by dissolving 60 mg of material in 0.7 mL of d_6-DMSO. The samples were then sonicated for 30 mins at 35°C and then filtered through a 0.45 µm PTFE syringe filter. All samples were allowed to thermally equilibrate prior to optimizing DOSY parameters (Δ and δ).

Extraction of Lignin

Dioxasolv,[S2] butanosolv[S3] and formadehyde stabilised[S4] lignins were extracted using previously reported procedures.

Gel Permeation Chromatography

Samples were prepared by dissolving lignin (ca. 7 mg) in pyridine (0.5 mL) and acetic anhydride (0.5 mL) and the solution was stirred for 16 hours. The mixture was concentrated in vacuo by azeotropic distillation with toluene (3x), ethanol (3x) and dichloromethane (3x). The residue was dissolved in THF (1 mL) and filtered through a 0.45 µm PTFE syringe filter and submitted for analysis. GPC analysis was carried out using a Shimadzu HPLC/GPC system equipped with a CBM-20A communication bus, DGU-20A degassing unit, LC-20AD pump, SIL-20A auto sampler, CTO-20A column oven and SPD 20A UV-Vis detector. Samples were analyzed using a Phenogel 5 µm 50A (300 x 7.8 mm) and Phenogel (5 µm 500A (300 x 7.8mm) columns connected in series and eluted with inhibitor free THF (1mL min-1) with a column oven temperature of 30°C. The system was calibrated using polystyrene standards sourced from Polymer Standards Services (PSS) with MP values ranging from 266 Da to 12600 Da.

Optimisation of co-solvent system

A flask was charged with lignin (300 mg) and 3 mL of acetone-methanol co-solvent at particular ratio. The solution was allowed to stir for an hour. The insoluble fraction was filtered off. The solvent was removed from the soluble fraction *in vacuo*. Yields of the soluble fractions were then compared.

Fractionation of lignin using acetone-methanol (3:2) solvent ratio

To lignin was added diethyl ether (10 volumes v/w) and the mixture was allowed to stir vigorously for 1 hour. The insoluble fraction was filtered off and dried under vacuum. The

filtrate was concentrated and dried *in vacuo*. This process was repeated, with addition of 5% increments of acetone/methanol (3:2) solution in diethyl ether solution until 100 % acetone/methanol (3:2) solution was used. After the last fractionation step, all the fractions were then dried in vacuo for a further 8 hours before being weighed.

Fractionation of Kraft lignin for Protocol comparison

Our fractionation (P1) and the fast fractionation protocol (P2) were both adapted so that 8 lignin fractions were generated.

Protocol P1

To Kraft lignin (20 g) was added a solution of acetone-methanol (3:2) co-solvent system (14%) in diethyl ether (86%,10 volumes v/w in total) and the mixture was allowed to stir vigorously for 1 hour. The insoluble fraction was filtered off and dried under vacuum. The filtrate was concentrated and dried *in vacuo*. This process was repeated, with addition of 14% increments of acetone/methanol (3:2) co-solvent solution in diethyl ether until 100 % acetone/methanol (3:2) solution was used (last step was rounded up to 100% acetone/methanol solution (3:2). After the last fractionation step, all the fractions were then dried in vacuo for a further 8 hours before being weighed.

Protocol P2

Kraft lignin (20 g) was stirred in a solution of acetone methanol (3:2, 200 mL). The insoluble material was filtered off and dried *in vacuo*. To the vigorously stirring soluble fraction was added, hexanes (20 mL). The solution was decanted off leaving a gummy residue on the side of the flask. The precipitated gummy residue was dried *in vacuo*. This process was repeated 5 times using 20 mL, , 40 mL, 70 mL and 100 mL in this order at each step. The remaining soluble material was concentrated and dried *in vacuo*. All the fractions were then dried in vacuo for a further 8 hours before being weighed.

References

[S1] Y. Y. Wang, M. Li, C. E. Wyman, C. M. Cai, A. J. Ragauskas, *ACS Sustain. Chem. Eng.* **2018**, *6*, 6064–6072.

[S2] Y. Ni, Q. Hu, *J. Appl. Polym. Sci.* **1995**, *57*, 1441–1446.

[S3] C. S. Lancefield, O. S. Ojo, F. Tran, N. J. Westwood, *Angew. Chem. Int. Ed. Engl.* **2015**, *54*, 258–62.

[S4] C. S. Lancefield, I. Panovic, P. J. Deuss, K. Barta, N. J. Westwood, *Green Chem.* **2017**, *19*, 202–214.

[S5] L. Shuai, M. T. Amiri, Y. M. Questell-Santiago, F. Héroguel, Y. Li, H. Kim, R. Meilan, C. Chapple, J. Ralph, J. S. Luterbacher, *Science (80-.).* **2016**, *354*, 329–333.
